# Serum Folate deficiency in HCV related Hepatocellular Carcinoma

**DOI:** 10.1038/s41598-022-09030-1

**Published:** 2022-03-23

**Authors:** Giulia Malaguarnera, Vito Emanuele Catania, Gaetano Bertino, Filippo Drago, Roberto Madeddu, Claudia Bonfiglio, Michele Malaguarnera

**Affiliations:** 1 Department of Biomedical and Biotechnological Science, University of Catania, Catania, Italy; 2 Medical, Surgical Sciences and Advanced Technologies “G.F. Ingrassia”, University of Catania, Catania, Italy; 3 Department of Experimental and Clinical Medicine, University of Catania, Policlinico “G. Rodolico”, Via S. Sofia n.78, 95123 Catania, Italy; 4 Department of Biomedical Sciences, University of Sassari, Catania, Italy; 5 Medical Centre INPS Catania-Italy, Catania, Italy

**Keywords:** Cancer metabolism, Cancer, Liver cancer

## Abstract

Nutritional and environmental factors had been reporting in the progression of hepatocellular carcinoma (HCC). In this study, we focused our intervention in the correlation between the folate status and the progression of HCC in patients with chronic virus C (HCV) infection. Nine-eight patients, HCV positive with HCC and one hundred of patients with HCV positive liver cirrhosis (LC) and one hundred patients with HCV positive chronic hepatitis (CHC) and one hundred control subjects were enrolled. The viremia for hepatitis C patients (HCV) was determined by HCV RNA with polymerase chain reaction. HCV was confirmed by HCV RNA or a positive anti-HCV test with chronic liver disease. The comparison of folate serum levels in HCC patients vs Liver Cirrhosis (LC) patients showed a significant decrease of 1.16 ng/ml *P* = 0.0006 (95% CI-1.925 to − 0.395), in HCC patients versus CHC a decrease of 1.40 ng/ml *P* < 0.0001 (95% CI-2.16 to − 0.63), in HCC vs controls a decrease of 3.80 ng/ml *P* < 0.0001 (95% CI-4.56 to − 3.03). The comparison of homocysteine Hcy serum levels showed a significant increase in HCC vs LC of 4 nmol/L (*P* < 0.0001, 95% CI 2.77 to 5.22) versus CHC of 9 nmol/L (*P* < 0.0001, 95% CI 7.78 to 10.22) and vs Controls 9.30 nmol/L (*P* < 0.0001, 95% CI 8.07 to 10.52). With progression of HCV infection from chronic hepatitis to cirrhosis, then to HCC development, serum folate levels are progressively decreasing together with a progressive increase in serum homocysteine levels reflecting its role in disease progress and carcinogenesis.

## Introduction

Hepatocellular carcinoma (HCC) is the most common form of liver cancer being diagnosed annually, being seventh in incidence and the fourth in mortality in the global cancer statistic.

Chronic infection with hepatitis B and C viruses, food contamination with aflatoxins, occupational exposure, excessive alcohol intake, diabetes mellitus, metabolic syndrome, cirrhosis, obesity, nutritional deficiencies, diet habits, smoking, sociodemographic characteristic, genetic factors are involved in the insurgence, development and progression of HCC.

Primary prevention of HCC can be achieved with vaccination against HBV infection. Recent advances with direct-active-antivirals (DAA) against HCV infection shows a potential effect in reduction of HCC incidence.

However, some reports claimed that HCC remain higher in patient after DAA therapy than after interferon-based treatment. Studies shows an increased risk of early tumour recurrence in treated patients with HCC who received DAA treatment^1,2^.

This condition requires new efforts to identify risk and new treatments that improve their effectiveness^3–9^.

Complex relationship between genetic, etiologic and environmental risk factors make difficult to identify the pathologic mechanisms of hepatocarcinogenesis, development and progression of HCC. The role of the folate in the transmethylation and transsulfuration involved in mechanism of homocysteine reduction and in methionine activity and may contribute to identify new prognostic markers and opportunities for target therapy. Nutritional deficiencies, including folate, Betaine and vitamin B_12_ and deficient intake of micronutrient involved in one-carbon metabolism in the development of HCC, may play an important role in the pathogenesis and progression of Hepatitis C to cirrhosis and to hepatocellular carcinomas. Particularly, folate deficiency is one of the commonest forms of vitamin deficiency, occurring in about 10% of the population^10^. Folate is a water-soluble beta-vitamin critically involved in the one-carbon metabolism and is essential for DNA methylation, synthesis, and repair during cell regeneration. Folate is not synthesized by the body, but derived from folate rich food (leafy vegetables, fruit, yeast, mushrooms, and animal proteins)^11,12^. Folate availability plays an important role in determining the integrity of one-carbon metabolism, derangements resulting in genetic and epigenetic changes related to carcinogenesis. An adequate folate status supplies the liver with available one carbon carriers for purine synthesis, amino acid interconversion and methylation in macromolecules^13–17^.

Based upon these considerations, we undertook the present study to determine the folate serum concentration in HCC patients with HCV and to postulate a possible the mechanistic effect of folate^18–20^.

## Materials and methods

### Patients

#### Patient selection

Ninety-eight consecutive patients HCV positive with HCC and one hundred patients with liver cirrhosis (LC) and one hundred patients with chronic hepatitis (CHC) were enrolled at the outpatient practice of the Centre of Senescence between October 2009 to June 2017. Exclusion criteria were also coinfection with hepatitis B and or Human Immunodeficiency Viruses; any anti-HCV therapy in the previous 12 months. We also excluded patients who had other major chronic condition, other malignancies, orthotopic liver transplantation and those are unable to consent.

Clinical information for patients diagnosed with hepatocellular carcinoma between December 2009 to April 2016 were extracted from medical records. For all studies groups, a detailed history, complete clinical assessment, biochemical liver, α-Fetoprotein, blood glucose level, hepatitis markers, HCV RNA, abdominal ultrasonography and triphasic CT scan were performed. Cirrhosis was established histologically or clinically or both. Disease severity included early cirrhotic patients (Child A) and advanced cirrhotic patients (Child B and C) according to a modified Child–Pugh classification^21,21^. Histological severity was determined for patients with chronic hepatitis, by using the histological activity index (HAI). Whereas the CHC patients were diagnosed by elevated liver enzymes for more than 6 months and imaging evidence of CHC, the LC group was diagnosed by abdominal ultrasonography and histologically. The diagnosis of hepatocellular carcinoma was based on histology, aspiration cytology, radiology or serum alpha-fetoprotein concentrations, including the presence of growing mass lesion in the liver detected by ultrasound, typical imaging features a cross-sectional contrast CT or MRI, an increase in serum alfa-fetoprotein to more than 200 ng/ml and the patients’ progressive clinical course. Alpha-fetoprotein (αFP) is the most used circulating marker for HCC. In our series, as in other, a small percentage of patients with HCC had αFP serum values > 200 ng/mL, a cut off considered suggestive of tumour^23,24^.

The level of viremia for patients with hepatitis C (HCV) was determined by HCV RNA with polymerase chain reaction (PCR, Amplicor HCV Monitor, Roche Molecular Systems, Branchburg NJ). HCV was confirmed by HCV RNA or a positive anti-HCV test with chronic liver disease.

The Barcelona-Clinic Liver Cancer (BCLC) were applied to HCC patients.

BCLC classifies the patient in 5 stages (0, A, B, C, D) according to the prognostic variables related to tumour status (size, number, vascular invasion, N + , M +), liver function Child–Pugh’s classification) and health status (ECOG)^25,26^. In cirrhotic patients’ nodules more 2 cm in diameter were diagnosed for HCC based on typical features on one imaging technique^27^.

#### Laboratory analyses

Blood was collected from an antecubital vein into two 4-ml vacuum-evacuated ETDA-containing tubes and one 10 ml tube that did not contain anticoagulants. Venous blood samples were drawn from patients and all examinations were performed at 8.00 h after an overnight fast. Laboratory data included aminotransferases, alkaline phosphatase, total bilirubin, serum albumin and prothrombin.

Total cholesterol, triglycerides and fasting plasma glucose were enzymatically measured (Roche/Hitachi 912 analyser: Roche Diagnostics, Switzerland). Plasma alpha-fetoprotein (AFP) was measured by automatic electrochemical immunalyzer (Roche Cobas E601). Serum creatinine levels were assayed with routine laboratory method. Serum was used for the analysis of folate and for the analysis of Hcy. All specimens were collected and analysed within 1 h or stored ad − 80 °C until use. Serum homocysteine were quantified using a high-performance liquid chromatography^28,29^. The precision of the assay was monitored by analysing pooled plasma in duplicate. Serum folate concentrations has been measured using Quantaphaseradioassay kit (Bio-Rad Laboratories). We calculated the proportion of participants with low serum folate concentrations, and we used the cut-off of 6.0 nmol/L to indicate low serum folate concentrations. The concentration of folate and Hcy were determined on the same day.

### Ethics approval and consent to participate

Study recruitment was performed in observation and respect of Helsinki Declaration. All patients gave their written informed consent for the study participation. The institutional review board of Centre of Senescence, University of Catania has approved the study.

### Patient consent for publication

All patients gave their written informed consent for the publication of the study.

## Statistical analysis

Data were expressed as mean + /− standard deviation. To assess relationship and differences between data, parametric and tests were performed (ordinary one-way ANOVA, Fisher exact test). P value < 0.05 was considered statistically significant. Fisher 's exact test, two-sided, with Baptista-Pike compute of Confidence Intervals, was used to identify association of the folate serum levels with various clinical pathologic features. Multiple comparisons were generated to assess the relationship between folate concentration and sociodemographic data, area od residence (urban, metropolitan, semiurban, rural) social class (managers, skilled, semiskilled unskilled) and clinical features of HCC patients. Data were analysed using the GraphPad Prism statistical software package (9.1.2 Macintosh Version; GraphPad Software San Diego, CA).

## Results

### Baseline characteristic

Baseline and Anthropometric characteristics of the patients in the study groups are shown in Table 1.

**Table 1:** Baseline and anthropometric characteristics of the patients.

	HCC	LC	CHC	Controls	HCC vs LC	HCC vs CHC	HCC vs Controls	LC vs CHC	LC vs Controls	CHC vs Controls
	98 pt	100 pt	100 pt	100 pt	*P*	*P*	*P*	*P*	*P*	*P*
Sex (F/M)	47/51	49/51	49/51	49/51						
Age (years)	49.50 ± 8.74	48.90 ± 9.05	49.10 ± 8.91	50.20 ± 8.78	0.636	0.75	0.575	0.875	0.304	0.38
Body Mass Index (BMI) (Kg/m2)	23.8 ± 2.3	24.8 ± 3.1	24.6 ± 3.0	24.7 ± 2.7	*0.011*	*0.037*	*0.012*	0.643	0.808	0.805
HCV exposure time (years)	16.8 ± 8.2	14.2 ± 6.4	10.4 ± 3.2	–	*0.014*	< *0.001*	–	< *0.001*	–	–
Viremia (U/L)	3.8 ± 2.8	4.2 ± 2.6	4.8 ± 2.4	–	0.299	*0.008*	–	0.092	–	–
**HCV genotype**										
*1a*	4	6	6	–						
*1b*	85	84	85	–						
*2a*	5	5	4	–						
*3a*	4	5	5	–						

Ordinary one-way ANOVA with Tukey's multiple comparisons test.

Summary: NS: non significative; *P* < 0.05 significative.

*CHC* chronic hepatitis C, *LC* liver cirrhosis.

The age of HCC patients, cirrhotic patients, CHC patients and subject control ranged between 40 to 75 years. BMI is decreased in HCC vs cirrhotic 1.00 kg/m^2^ (*P* = 0.01) vs hepatitis 0.8 (*P* = 0.037) vs controls 0.9 (*P* = 0.012). (Table 1).

The 28.57% of HCC subjects were Child–Pugh class A, 53.06% were class B, 18.37% were class C. Cirrhosis Child class A in HCC vs Liver cirrhosis patients is decreased *P* = 0.02; no significant differences were found in child class B and in Child class C. HCV exposure is increased in HCC vs cirrhosis 2.6 years (*P* = 0.01) vs hepatitis: 6.4 years (*P* < 0.001); viremia in chronic hepatitis vs HCC is increased 1.0 in mean IU/L (*P* = 0.008) (Table 2).

**Table 2:** Risk factors of HCC patient at baseline.

	HCC		LC		CHC		Controls		LC vs CHC		LC vs Controls		CHC vs controls		HCC vs LC		HCC vs CHC		HCC vs controls	
	98 pt	%	100 pt	%	100 pt	%	100 pt	%	*P-value*	*	*P-value*	*	*P-value*	*	*P-value*	*	*P-value*	*	*P-value*	*
Alcoholic habit +	25	25.51	36	36.00	44	44.00	48	48.00	0.3123	NS	0.1148	NS	0.6705	NS	0.6988	NS	0.0074	**	0.0012	**
Alcoholic habit -	73	74.49	64	64.00	56	56.00	52	52.00												
Smoking habit +	36	36.73	41	41.00	44	44.00	42	42.00	0.7749	NS	> 0.9999	NS	0.8865	NS	0.5625	NS	0.3140	NS	0.4701	NS
Smoking habit -	62	63.27	59	59.00	56	56.00	58	58.00												
Cirrhosis Child A	28	28.57	44	44.00	/	/	/	/	/	/	/	/	/	/	0.0272	*	/	/	/	/
Cirrhosis Child B	52	53.06	40	40.00	/	/	/	/	/	/	/	/	/	/	0.0870	NS	/	/	/	/
Cirrhosis Child C	18	18.37	16	16.00	/	/	/	/	/	/	/	/	/	/	0.7088	NS	/	/	/	/
Diabetes absence	38	38.78	40	40.00	56	56.00	71	71.00	0.0335	*	< 0.0001	****	0.0394	*	0.8852	NS	0.0163	*	< 0.0001	****
Diabetes presence	60	61.22	60	60.00	44	44.00	29	29.00												

Fisher 's exact test, 95% CI Odds Ratio (O.R.) Baptista-Pike method.

Summary: NS: non significative; * < 0.05; ** < 0.005; *** < 0.001; **** < 0.0001.

Cirrhosis Child class A showed OR = 0.51, *P* = 0.02 (95% CI 0.28 to 0.90 CI), alcoholic consumption shows in CHC OR = 0.44 *P* = 0.007 (95% CI 0.24 to 0.80) and in controls OR 0.37 *P* = 0.0012 (95%CI 0.20 to 0.67). Diabetes mellitus presence in CHC showed OR 0.45 *P* = 0.01 (95% CI 0.29 to 0.88) and in controls OR = 0.26, *P* < 0.0001 (95% CI 0.14 to 0.47). (Table 2).

#### Comparison of biochemical tests in the cohort groups

The comparison between HCC to Liver Cirrhosis (LC) patients showed higher mean value of ALT (*P* < 0.001), AST (*P* < 0.001), γGT (*P* < 0.0001), HDL Cholesterol (*P* = 0.0004), Triglycerides (*P* < 0.0001), α-Fetoprotein (*P* < 0.001), in INR (*P* < 0.001), and a lower mean value of Total Cholesterol (*P* < 0.0001), and fasting glucose (*P* < 0.0001).

The comparison between HCC and CHC subjects showed a higher mean value in ALT (*P* = 0.01), in AST (*P* < 0.001), in γGT (*P* < 0.0001), in Total Bilirubin (*P* < 0.001), in α-Fetoprotein (*P* < 0.001), in BUN (*P* < 0.0001), in INR (*P* < 0.001), and a lower mean value in Total Cholesterol (*P* < 0.0001), in albumin(*P* < 0.001), in fasting glucose (*P* < 0.0001) and in serum creatinine (*P* = 0.0334).

Comparison between HCC and Controls subjects showed a higher mean value of ALT (*P* < 0.001), AST (*P* < 0.001), γGT (*P* < 0.0001), α-Fetoprotein (*P* < 0.001), in INR (*P* < 0.001), in Creatinine (*P* < 0.0001), in total Bilirubin (*P* < 0.001) and a lower mean value of total Cholesterol (*P* < 0.0001), Triglycerides (*P* < 0.0001), in Albumin(*P* < 0.001), in fasting Glucose (*P* < 0.0001) and in BUN (*P* = 0.0012).

Comparison between LC and CHC patients showed a higher mean value of ALT (*P* < 0.001), AST (*P* < 0.001), γGT (*P* < 0.0001), α-Fetoprotein (*P* < 0.001), in total Bilirubin (*P* < 0.001), in INR (*P* < 0.001), in BUN (*P* = 0.0011) and a lower mean value of Triglycerides (*P* < 0.0001) and in Albumin (*P* < 0.001).

Comparison between LC and Controls subjects showed a higher mean value of ALT (*P* < 0.001), AST (*P* < 0.001), γGT (*P* < 0.001), in serum Creatinine (*P* < 0.0001), and a lower mean value of total Cholesterol (*P* < 0.0001), HDL Cholesterol (*P* = 0.003), Triglycerides (*P* < 0.0001), in Albumin(*P* < 0.001), in α-fetoprotein (*P* < 0.001), and in INR (*P* < 0.001).

Comparison between CHC and Controls subjects showed a higher mean value of ALT (*P* < 0.001), AST (*P* < 0.001), in Total bilirubin (*P* < 0.001), and a lower mean value of total Cholesterol (*P* = 0.0005), in Triglycerides (*P* < 0.0001), in Albumin (*P* < 0.001), in α-Fetoprotein (*P* = 0.016), in serum Creatinine (*P* = 0.0005). (Table 3).

**Table 3:** Comparison of Biochemical test.

	HCC	LC	CHC	Controls	HCC vs. LC	HCC vs. CHC	HCC vs. Controls	LC vs. CHC	LC vs. Controls	CHC vs. Controls
	98 pt	100 pt	100 pt	100 pt	*P*	*P*	*P*	*P*	*P*	*P*
Alanine Transaminase (ALT) [IU/l] (n.v. 8–40)	78.40 ± 54.10	47.80 ± 29.20	62.40 ± 29.80	20.40 ± 8.10	< *0.001*	*0.01*	< *0.001*	< 0.001	< *0.001*	< *0.001*
Aspartate Transaminase (AST) [IU/l] (n.v. 8–40)	125.40 ± 55.10	61.40 ± 31.80	59.80 ± 36.40	24.20 ± 7.90	< *0.001*	< *0.001*	< *0.001*	< 0.001	< *0.001*	< *0.001*
γGT [IU/L] (n.v. 6–40)	94.5 ± 12.22	64.1 ± 10.8	52.1 ± 9.7	36.4 ± 8.7	< *0.0001*	< *0.0001*	< *0.0001*	< *0.0001*	< *0.0001*	< *0.0001*
Total Cholesterol mg/dL (n.v. < 200)	204 ± 10.8	212.1 ± 10.9	232.2 ± 10.8	238.1 ± 9.7	< *0.0001*	< *0.0001*	< *0.0001*	< *0.0001*	< *0.0001*	*0.0005*
HDL Cholesterol mg/dL (n.v. 40–60)	43.70 ± 5.2	40.10 ± 6.4	41.8 ± 6.5	43.20 ± 6.9	*0.0004*	*0.1466*	*0.9439*	*0.2246*	*0.003*	*0.3944*
Triglycerides mg/dL (n.v. < 150)	187.1 ± 3.6	174.6 ± 9.7	188 ± 9.6	196 ± 7.8	< *0.0001*	*0.8618*	< *0.0001*	< *0.0001*	< *0.0001*	< *0.0001*
Total bilirubin [mg/dL] (n.v. 0.2–1.2)	3.22 ± 1.24	2.97 ± 1.20	1.45 ± 1.37	0.91 ± 0.44	0.151	< *0.001*	< *0.001*	< 0.001	< *0.001*	< *0.001*
Albumin (g/dL) mg/dL (n.v. 3.5–5.0)	2.96 ± 0.54	2.87 ± 0.66	3.84 ± 0.76	4.51 ± 0.38	0.295	< *0.001*	< *0.001*	< 0.001	< *0.001*	< *0.001*
αFetoprotein [ng/ml](n.v. < 6.0)	536.10 ± 196.40	13.80 ± 10.20	5.10 ± 4.81	3.91 ± 0.80	< *0.001*	< *0.001*	< *0.001*	< 0.001	< *0.001*	*0.016*
Serum Folate (nm/L) (n.v. 6–45)	4.22 ± 1.87	5.38 ± 2.10	5.62 ± 1.93	8.02 ± 2.40	< *0.001*	< *0.001*	< *0.001*	0.401	< *0.001*	< *0.001*
Homocysteine (μmol/L) (n.v. < 13)	19.40 ± 3.20	15.40 ± 3.80	10.40 ± 3.20	10.10 ± 3.10	< *0.001*	< *0.001*	< *0.001*	< 0.001	< *0.001*	0.502
INR (n.v. 0.9–1.2)	2.60 ± 0.60	1.90 ± 0.70	1.00 ± 0.32	0.98 ± 0.30	< *0.001*	< *0.001*	< *0.001*	< 0.001	< *0.001*	0.649
Fasting glucose [mg/dL] (n.v. 74–106)	74.8 ± 9.2	81.4 ± 8.7	83.6 ± 7.4	83.9 ± 7.2	< *0.0001*	< *0.0001*	< *0.0001*	*0.2273*	*0.1348*	*0.9938*
Blood Urea Nitrogen [mg/dL] (n.v. 6–25)	45.1 ± 3.9	44.1 ± 4.2	41.8 ± 4.8	42.8 ± 4.3	*0.3625*	< *0.0001*	*0.0012*	*0.0011*	*0.1452*	*0.3579*
Serum Creatinine [mg/dL] (n.v. 0.7–1.3)	*0.96* ± *0.21*	*0.94* ± *0.22*	*0.87* ± *0.21*	*0.74* ± *0.28*	*0.9299*	*0.0334*	< *0.0001*	*0.1442*	< *0.0001*	*0.0005*

Ordinary one-way ANOVA (one for each row) with Tukey's multiple comparison test.

Summary: NS: non significative; *P* < 0.05 significative.

*CHC* chronic hepatitis C, *LC* liver cirrhosis.

### Comparison of folate and homocysteine in the cohort group

The comparison of folate serum levels in HCC patients versus (vs) cirrhotic patients showed a significant decrease of 1.16 ng/ml *P* = 0.0006 (95% CI-1.925 to − 0.395), in HCC patients vs CHC a decrease of 1.40 ng/ml *P* < 0.0001 (95% CI-2.16 to − 0.63), in HCC vs controls subject displayed a decrease of 3.80 ng/ml *P* < 0.0001 (95% CI-4.56 to − 3.03).

The comparison of Hcy serum levels showed a significant increase in HCC patients vs cirrhotic patients of 4 nmol/L *P* < 0.0001 (95% CI 2.77 to 5.22), versus CHC 9 nmol/L *P* < 0.0001 (95% CI 7.78 to 10.22), and vs controls 9.30 nmol/L *P* < 0.0001 (95% CI 8.07 to 10.52). The median value of serum folate levels was 5.6 nmol/L.

Comparison of Folate and Homocysteine serum levels in subgroup of HCC, in accordance with BCLC staging system.

In accordance with BCLC patients we classified 18 in stage 0, 20 in stage A, 25 in stage B, 20 in stage C and 15 in stage D. The comparison with HCC patients in stage 0 showed a significant decrease of serum folate level in HCC patients in stage C of 4.8 ng/ml (95% CI 0.4929 to 9.107) *P* = 0.02 and more in patient in stage D of 5.7 ng/ml (95% CI 1.065 to 10.33) *P* = 0.0076.

The comparison with patient in stage 0 showed a significant increase in serum Homocysteine levels in patients in stage B of 13.5 nmol/L (95% CI-17.60 to − 9.402) *P* < 0.0001, in stage C of 15.3 nmol/L (95% CI-19.61 to − 10.99) *P* < 0.0001 and more in stage D, with 26.5 nmol/L of difference (95% CI-31.13 to − 21.87) *P* < 0.0001.

In the present study, the low serum folate levels were not associated in multiple comparison with changes in BMI, levels of albumin, AST, ALT. A significant negative correlation between folate concentration and Hcy level (0.005 R = − 0.40) was observed.

## Discussion

Liver is a central organ for many metabolic and synthetic functions and stores essential nutrients as folate, which is involved in the methylation of homocysteine. HCV infection can lead with mitochondrial dysfunction and hepatocyte oxidative stress, to the development of HCC. Whilst folate deficiencies may be mediated by HCV replication and may be further exacerbated by malnutrition^30^.

The HCV is one of the major risk factors for HCC, as categorized by the International Agency for Research on Cancer (IARC). HCV induce hepatocarcinogenesis, involving the activation of multiple cellular pathways and gets initiated by HCV infection leading to chronic hepatic inflammation, which progress to liver cirrhosis and HCC. HCC development in HCV is associated with fibrosis and the viral colony number^31^. In our study we observed that HCV exposure time is higher in HCC than LC and CHC (Table 1).

No significant differences were observed between genotypes and viremia (Table 1).

HCV proteins have been shown to promote cellular proliferation, transformation, and tumour growth. Over-expression of HCV core proteins, NS3 and NS5A, inhibit tumour suppressor genes, E2 and NS5B activate the RAF/mitogen activated protein Kinase (MAPK) / ERK pathways, NS5A activates the PI3K/AKT and beta-catenin/WNT pathways and evades apoptosis by caspase-3-inhibition^32,33^.

Hepatocellular carcinoma is the result of underlying and well-defined liver disease in most patients.

Awareness of rising incidence of HCC and of insufficient knowledge of hepatopathy, is critical to prevent, detect and treat liver cancer.

HCC is characterized by abundant phenotypic and molecular alterations, which are not always translated into improved prognostic assessment or therapeutic decision making.

**Figure 1:**
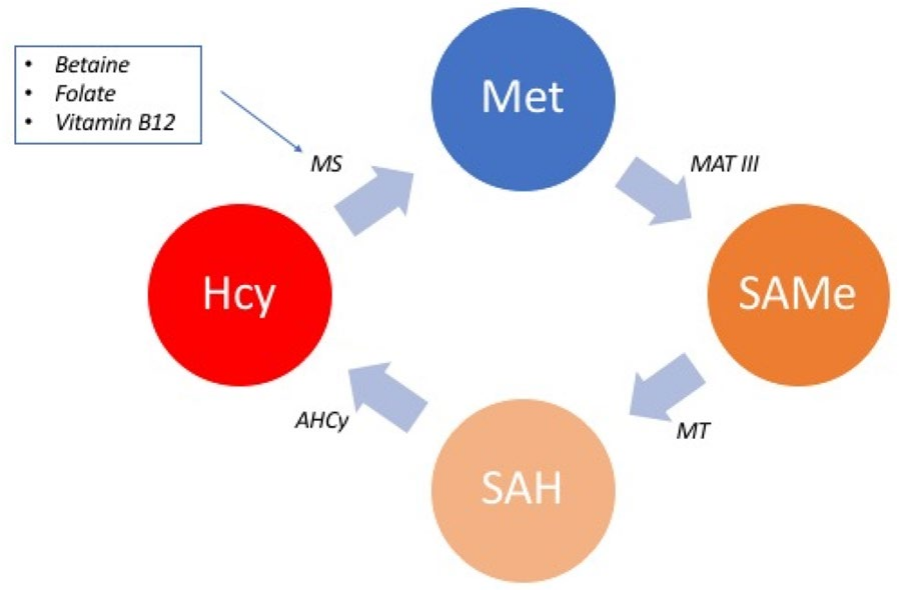
Methionine cycle. (clockwise) MS: Methionine Synthase; Met: Methionine; MAT III Methionine adenosyl-transferase; SAMe s-adenosyl-methionine; MT: Methyl Transferase; SAH: s-adenosyl-homocysteine; AHCy: s-adenosyl-homocysteine hydrolase; Hcy: homocysteine.

Liver lesions, induced by virus, fibrosis, cirrhosis, xenobiotics, including preneoplastic and neoplastic alterations, are associated with modifications of the methionine cycle, involved in transmethylation and in transsulfuration pathway (Fig. 1).

Folate deficiency favours the progression of liver disease through mechanisms that include its effects on methionine metabolism. Cirrhosis is an important risk factor for HCC and may be caused by chronic viral hepatitis, Alcoholic liver disorders, non-alcoholic fatty liver diseases, metabolic diseases.

Risk factors of HCC are the presence of alcohol habit, metabolic syndrome, smoking and cirrhosis. The presence of alcohol consumption causes poor absorption causing oxidative damage and affect DNA methylation pathways by promoting the degradation and inhibiting the metabolism of this nutrient^34,35^. Some studies failed to show a significant positive association between alcohol consumption and HCC. In fact, the risk depends on the quantity and duration of alcohol consumption which act synergically or in addition to factors such as gender, age, presence of metabolic syndrome.

The link between cigarette smoking and HCC was conflicting from no significant to a threefold increase compared to no smokers^36^. Cigarettes contain carcinogen components, such as arsenic, aromatic hydrocarbons, diethyl nitrosamine, 4-aminobiphenyl, vinyl chloride, 4-aminophenil and N-nitroso-dimethylamine, which may contribute to liver carcinogenesis. The tobacco abuse can progress the development of CHC to cirrhosis and to HCC. Moreover, the tobacco may cause the dysfunction of genes as well the enzymes involved in the detoxification of nicotine^37,38^ (Table 2).

In this study we observed a significant decrease of serum folate levels in HCC, in LC and in CHC.

**Figure 2:**
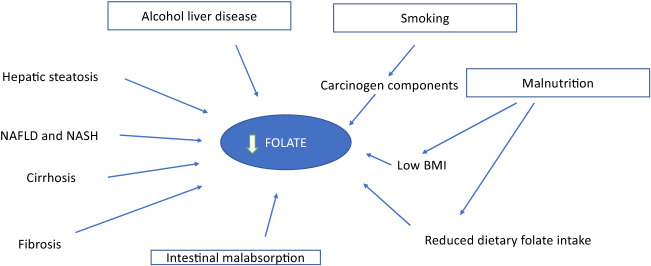
HCC risk factors and folate deficiency.

The mechanism of folate deficiency is multifactorial and involve several pathways (Fig. 2).

Age, Alcoholic liver disease, malnutrition, intestinal malabsorption, reduced liver uptake, reduced storage and increased urinary folate excretion favours the deficiency and consequently favours the progression of liver disease. Alcohol consumption can alter metabolic pathways including fatty acid oxidation and lipogenesis. Chronic alcohol consumption leads to an abnormal accumulation of acetaldehyde, which can exert carcinogenic effects through the formation of DNA–protein adduct^39^.

The decrease of serum folate in HCC patients may have resulted from several factors as chronic alcohol consumption, malnourishment, malabsorption, or the severe catabolic status^40,41^. Folate plays an important role in DNA synthesis, repair and methylation. Low plasma folate concentrations may increase the risk of HCC by inducing DNA hypomethylation, leading to dysregulation of proto-oncogenes and tumour suppressor genes^42,43^. Deficiency or impairment of folate metabolism is associated with high levels of homocysteine, hypomethylation (the decreased one-carbon unit transfer to purines and pyrimidines for DNA repair and biosynthesis), DNA damage, and impaired cell proliferation, malignancies and impaired NO production^45,46^.

Cirrhosis is the consequence of long-term liver damage with functional alterations due to chronic inflammation. The majority of HCC occurs in cirrhotic patient, and between 5 and 30% of these patients develop HCC within the first five years^47^. Patients with liver cirrhosis have diminished folate reserves, as water soluble vitamin, not only for hepatic dysfunction, for low dietary intake, for low absorption, for increased catabolism but also for use of diuretics^48^ (Table 2).

Cirrhosis may develop HCC during their lifetime (2% in HBV infected cirrhosis and 3–8% in HCV-infected cirrhosis)^49^. We observed higher Hcy serum levels and lower folate serum levels in HCV-related HCC than in patients with liver cirrhosis, chronic hepatitis and in the control subjects.

Biochemical findings showed a significant increase in transaminases, γ-GT, bilirubin, α fetoprotein, triglycerides in HCC associated with increase of Homocysteine and a decrease in folate and albumin, INR (Table 3).

These biochemical finding associated with clinical symptoms (ascites, encephalopathy, portal hypertension) are a important expression of hepatic decompensation. Malnutrition is common in chronic liver diseases and increases the severity of liver disease in patient with liver cirrhosis. The prevalence of malnutrition varies depending on diseases severity: it occurs in about 20%v of patients with compensate disease and up to 80% of patients with severe liver failure^48,50^ (Table 2).

Reduced dietary intake, altered metabolism and malabsorption play an important role in the pathogenesis of malnutrition in cirrhosis and HCC. The functions of inflammation and pro-inflammatory cytokines with associated anorexia help to sustain bodily functions in the face of infection and injury. Ascites is a common symptom of hepatic decompensation that directly impacts oral intake by limiting physical capacity of the stomach and indirectly by contributing to post-prandial discomfort. Hepatic encephalopathy and sarcopenia are closely related to malnutrition, sharing common etiological factors and pathological pathways linked to muscle health in liver disease^51^. Recent studies have demonstrated that the incidence of HCC in patients with NASH ranges from 2.4% over the span of 7 years to 12.8% over the span of 3 years^52^.

In HCC patients, classified with BCLC system we observed that folate serum levels were lower both in stage C and in stage D than in stage 0, A and B; Homocysteine were higher in B, C, and D than in A (Table 4).

**Table 4:** Multiple Comparison of Means and Standard Deviations of Folate and Hcy Values by Stage.

	0			A			B			C		
	18			20			25			20		
	95% CI	*	*P*	95% CI	*	*P*	95% CI	*	*P*	95% CI	*	*P*
**Comparison of Folate**												
vs A	− 2.807 to 5.807	ns	0.8729	/	/	/	/	/	/	/	/	/
vs B	− 0.7980 to 7.398	ns	0.1775	− 2.177 to 5.777	ns		/	/	/	/	/	/
vs C	0.4929 to 9.107	*	0.0205	− 0.8922 to 7.492	ns	0.1964	− 2.477 to 5.477	ns	0.8369	/	/	/
vs D	1.065 to 10.33	**	0.0076	− 0.3281 to 8.728	ns	0.0832	− 1.930 to 6.730	ns	0.5463	− 3.628 to 5.428	ns	0.9821
**Comparison of Hcy**												
vs A	− 7.707 to 0.9071	ns	0.194	/	/	/	/	/	/	/	/	/
vs B	− 17.60 to − 9.402	****	< 0.0001	− 14.08 to − 6.123	****	< 0.0001	/	/	/	/	/	/
vs C	− 19.61 to -10.99	****	< 0.0001	− 16.09 to − 7.708	****	< 0.0001	− 5.777 to 2.177	ns	0.7239	/	/	/
vs D	− 31.13 to − 21.87	****	< 0.0001	− 27.63 to − 18.57	****	< 0.0001	− 17.33 to − 8.670	****	< 0.0001	− 15.73 to − 6.672	****	< 0.0001

Two-way ANOVA with Tukey's multiple comparisons test.

*Summary:NS: not significative; * < 0.05; ** < 0.005; *** < 0.001; **** < 0.0001.

The measurement of plasm homocysteine provides a sensitive functional biomarker of folate status, but homocysteine is not a specific marker of folate status, because it should also be elevated with other B vitamin deficiencies, lifestyle factors renal insufficiency and cirrhosis. Plasma homocysteine is inversely related to folate. It is also highly responsive to intervention with folate, alone otr in combination with other methyl donors involved in one carbon metabolism, as vitamin B12, B6, riboflavin, betaine, choline or carnitine^53,54^.

Folate decrease include homocysteine remethylation and consequently reduction of SAM concentrations and elevation in plasma homocysteine and s-adenosylhomocysteine (SAH) concentration, leading to hypomethylated DNA and protein (including histones which affect gene expression and DNA stability (Fig. 1).

When the folate is low or deficient, plasma homocysteine is invariably found elevated.

The folate cycle coupled with the methionine cycle, constitute a double ring metabolic pathway. Folate comprises a network of interconnected folate-dependent metabolic pathways responsible for serine e glycine interconversion, de novo purine synthesis, de novo thymidylate synthesis and homocysteine remethylation to methionine^55^. Low folate status beyond a certain threshold may result in mitochondrial dysfunction and ROS-generated vicious cycles leading to apoptotic signaling and cellular death, all of which constitute a carcinogenic mechanism^56^.

Free methionine is absorbed and used for protein synthesis or is converted to S-adenosylmethionine (Fig. 1) by the enzyme methionine adenosyl-transferase (MAT), using ATP as co-substrate. SAMe’s methyl group is transferred to substrates, such as DNA, RNA, proteins, glycine and is converted to adenosylhomocysteine (SAH) which is the by-product of all transmethylation reaction and is an inhibitor of MAT^57^. SAMe acts as a major methyl and sulphate group donor in numerous biochemical reactions and may improve liver function and have a positive influence on liver diseases^57^. Methionine metabolism is closely related to various metabolic pathways.

Both dietary and endogenous folate play a significant role in hepatic metabolism, which in turn regulates homocysteine levels, antioxidant defenses, DNA assembly, lipid export and all epigenetic methylation reactions that contribute to gene expression regulation. Methionine metabolism is the pivot linking the folate cycle to the trans-sulphuration pathway. Homocysteine is a sulphur-containing, non-proteic, toxic amino acid, connects the trans-sulphuration pathway, with methionine cycle (Fig. 1).

Limitation of this study is that it is conducted in a single-center and has a relatively small sample size. Our analyses were based on single measurement of nutrients involved in the one-carbon metabolism at baseline, assuming stable serum concentration during follow up. Blood concentration may differ over time because of day-to-day variation and long-term changes within persons. Finally, we did not have information on polymorphisms in MTHF or other genes which play an important role in DNA methylation. Therefore, it is difficult to determine whether changes in serum folate levels were a risk factor for HCC development.

## Conclusion

Given that the liver is responsible for the storage and metabolism of many micronutrients, chronic HCV replication has the potential to negatively influence the folate states.

With progression of HCV infection from chronic hepatitis to cirrhosis, then to HCC development, serum folate levels are progressively decreasing together with a progressive increase in serum homocysteine levels reflecting its role in disease progress and carcinogenesis.

Future longitudinal study based on the baseline serum folate levels will be necessary.

Future challenges in the understanding of folate functions in liver pathophysiology will include issue such as the delineation of the precise molecular events through which this compound regulates gene expression.

## Data Availability

Please contact the authors for data requests.
